# Innovation strategy on the example of companies using bamboo

**DOI:** 10.1186/s13731-020-00144-2

**Published:** 2021-01-11

**Authors:** Piotr F. Borowski

**Affiliations:** Institute of Mechanical Engineering, Warsaw University of Life Sciences, 166 Nowoursynowska str., 02-787 Warsaw, Poland

**Keywords:** Strategy of innovation, Bamboo, Management, Business economy, Environment, Clean energy, Mechanical processes, Eco-efficiency

## Abstract

Innovations and new technologies allow companies to function, work, and develop in an ever-changing environment. The article discusses the importance of innovative strategies and presents the results of research carried out on the role of each employee group (CEO, R+D department, other workers) in implementing innovations, depending on the size of the enterprise: micro company, mini company, medium company, and large company. A look not only through the prism of the size of the organization, but also by the groups of people (knowledge group) responsible for innovation is a novelty of the research and fills the gap in research on innovation of enterprises. Moreover, as an exemplification of theory which is used in practice, the article also presents innovations related to bamboo use in many enterprises from different sectors of the economy (energy, automobile, and textile). Bamboo, thanks to its mechanical and chemical properties, can become an innovative material widely used by various companies. Innovations based on the use of bamboo become eco-innovations that support eco-efficiency and the circular economy. The cognitive and utilitarian value of completed research lies in the possibility of a broad look at the innovation strategy (including bamboo as an innovative material) and in the possibility of its implementation and application in various enterprises operating on the market.

## Introduction

The strategy of innovation can be described as a plan of company activities and development in order to encourage, mobilize, motivate, and achieve advancements in technology or service by investing financial and human assets in research and development activities. Many researchers emphasize the existence of a positive impact of innovation on the financial and operational dimensions of business performance and indicate significant differences in the results generated depending on the type of innovation (Expósito & Sanchis-Llopis, 2019) in the scale of the enterprise and the entire economy (Mtar & Belazreg, 2020). Product innovations are strongly linked to company performance, while process, marketing, and organizational innovations are less related to productivity (Bartelsman, Falk, Hagsten, et al., 2019). The innovation process depends also on the size of enterprises (Pertuz & Pérez, 2020). All innovations depend on the knowledge. Innovation and entrepreneurship (Schumpeter combined these concepts) depend primarily on innovative people, their skills, and knowledge (Henning & McKelvey, 2020). According to Malerba and McKelvey, those people (employees) create knowledge-intensive innovative entrepreneurship (Malerba & McKelvey, 2020). Innovation strategy means taking key, pivotal, forward-looking activities regarding the implementation of a new product, service, marketing method, process, etc., into the market or selected industry that will be able to meet previously unmet needs of buyers or meet these needs in a new way (Hartmann, Reymen, & Van Oosterom, 2008; Sjödin & Kristensson, 2012). The innovation strategy defines the long-term goals, ways, and scope to which innovations (product, process, or organizational) will be used to build a strategic advantage. Creating innovation within a company is not an easy or quick task (Pisano, 2019).

The concept of strategy implies a long-term action plan, but we also can look at innovative activities related to some products or services as activities carried out in a shorter period (Innovation BIS 2025 - The Bank’s new medium-term strategy, 2020). As was noticed by Moore, in order to achieve the long-term innovation success, we also should focus on the middle term (Moore, 2007).

The ability to successfully implement new ideas consists of a whole range of competencies and skills—from market analysis to project and change management. An effective strategy is to show the ways of creating the uniqueness of the company so that it stands out among competing entities operating on the market. The changing market environment forces enterprises to constantly change and adapt to the requirements and expectations and creates the need for innovation. The companies are looking for new, innovative solutions (De Flander & Rovers, 2009; Shergian & Immawan, 2015; Suhaily, Khalil, Nadirah, & Jawaid, 2013). An unprecedented level of economic change dynamics and turbulence of the business environment motivates companies to implement innovation in business models (Bereznoy, 2019). The conducted quantitative and qualitative research indicates that some changes outperform others at an increasing pace, which generates many new gaps in the company’s operation. Often, innovation is associated only with a product or technology. And yet innovation is divided into product, process, organizational, marketing, or social. Therefore, any organization that does not directly create new products or technologies can also be included in innovative organizations.

### The difference between innovation and innovative

When conducting research on innovation and innovative, it is worth paying attention to both concepts, because in the literature on the subject, there is a noticeable difference in the understanding of both concepts.

Innovative, as defined by the OECD (Organization for Economic Co-operating and Development), is the use of new or significantly improved products (goods, services) or processes (OECD, 2020). Innovative also means new marketing methods or new organizational methods used in business practice, organizing jobs, or new external relationships, links, and associations. The concept of innovation is also important, seen as a process that results in innovation. The previously unknown product of a given company is the result of its innovation. If implemented in practice, it will become an innovation. Therefore, innovation can be understood and seen as the pursuit of innovation. Innovative is the skill and efficiency in realizing and implementing innovations. Innovative can also be described as the ability and self-motivation of an enterprise to continually search for and use in practice scientific research, new concepts, ideas, and inventions.

### Types of innovation

The concept of innovation is derived from the Latin word *innovatio* meaning renewal. Very often, the concept of innovation is also referred to the Latin word *novus*, which is new. The dictionary of foreign words defines innovation as introducing something new, newly implemented thing, newness, and reform. Because the concept of innovation is understood in various ways among economists, managers, and practitioners, here there are some synthetic definitions:

- *Innovation*—innovative action in industry or services both in relation to products (by creating new or significantly modifying existing ones) and in relation to processes through their improvement.
- *Innovation*—a change in the existing economic system, consisting of the development and implementation of new solutions for an enterprise and improvement of existing ones, aimed at increasing the effectiveness of its functioning, and thus the purposefulness of operation, technical, and production advantage as well as an economic advantage. It can also be creating a completely new system in the form of an enterprise (Berliński, 2003).
- *Innovation*—this is something new that reduces costs, reduces risk, or provides an improved product, service, and instrument that better meet the demand of market participants.

Simon H. (laureate of the Nobel Prize in Economics in 1978 and the Turing Award in 1975) on the basis of research conducted on a group of medium and small German enterprises showed that innovation is the main factor affecting success and the acquisition and retention of a key company market position. Enterprises that have achieved market leadership have been highly innovative (Kheirandish & Mousavi, 2018). Freeman Ch. defined the concept of innovation as the first commercial launch of a new product, process, or device on the market. Freeman also introduced into the literature a very apt, accurate statement: “do not introduce innovation, it means dying”, innovations are new things that attract customers to their lack, causes that the market loses interest in the company and the company slowly disappears among the competitions (Freeman, 1997). Peter Drucker also believed that innovation is any newly created product, a system that is the result of inspiration and work (Drucker, 2014). In addition to definitions derived from management specialists, it is worth quoting some definitions of innovation from business practitioners’ point of view (Table 1).

**Table 1:** Definition of innovation from business practitioners

Definition	Author’s name	Who is the author
Transforming ideas and concepts in a solution that will bring benefits and specific value to the client	Nick Skillicorn	Chief editor of Idea to Value and also the CEO & Founder of *Improvides Innovation Consulting*
The application and implementation of ideas and concepts that are innovative and beneficial. The beginning of all innovation, the main core of novel concepts is the employees’ creativity, the ability to create and generate new and advantageous ideas	David Burkus	Best-selling author, award-winning podcaster, and associate professor of management at Oral Roberts University
A fundamental, essential, and basic way in which a company adds solid and systematically growing value to its business and satisfy the needs of its customers and, consequently, to the business owners	Paul Hobcraft	Adviser of numerous organizations on innovation for over 15 years and is consistently considered one of the world’s top innovation bloggers

Source: own elaboration based on https://www.ideatovalue.com/

Summarizing, the most important elements listed in the definitions indicate, that the most significant, often highlighted elements related to innovation are an idea, implementation of an idea, taking up a real challenge, increasing the value of the company and customers.

Another typology in the perception of innovation comes from the statistical office (https://stat.gov.pl/en/metainformations/glossary/terms-used). In statistical surveys conducted by the Central Statistical Office, the concept of innovation covers all possible degrees of novelty: from new products and processes on a global scale (so-called absolute innovations), through new products and processes on the scale of the country or market on which the enterprise operates, to products and processes new only for a given enterprise, but already implemented in other enterprises, fields of activity, or countries (so-called imitative innovations).

A different division of innovations can be made depending on the depth of changes made and implemented and the time needed to implement them. Innovations can be divided into continuous and step (breakthrough) innovations. Continuous innovation means small changes or improvements in a process or product (its improvement, improvement to meet the needs of customers) that cause gradual progress in the enterprise. These are innovations that rely on the implementation of existing solutions for organizational, production, and technical processes. The goal is to improve technology and products based on the resources and knowledge of the enterprise.

In contrast, step innovations are more radical and innovative innovations that take the company to a higher completely changed level of functioning. The basis of the step innovation strategy is the launch of new products on the market, and its basis is a huge expenditure on research and development. For the company, it is a revolution that creates the opportunity to attract new buyers on the current market as well as in new market segments. This kind of innovation is considered revolutionary, creative, basic. The radicals are related to innovation management practices (Oke, 2007). The strategy of groundbreaking innovation often depends on a combination of innovative technologies and a high level of adaptability. The company should therefore develop such a strategy for creating and planning innovation that would consist of seeking development opportunities in gaining new results and investing in knowledge and improvement of employees, development of markets, new technologies, new products, modern equipment, and comprehensive service. It can be assumed that an innovation-oriented enterprise is one that has the following features:

- Conduct research and development (or purchases R&D projects in a relatively wide range);
- Transfer significant financial resources to this activity;
- Permanently implement new scientific and technical solutions;
- Has a large share of novelties (products, technologies) in the volume of production (services);
- Constantly innovate in the markets.

Nowadays, when the environment is exploited too quickly by human activity, eco-innovation plays an important role. According to UN, the eco-innovation is a new business approach which promotes and supports sustainable development throughout the entire life cycle of a product, while also boosting a company’s performance, competitiveness, and innovation (UN environment programme, Eco-innovation, 2020). In a similar vein, the European Commission speaks, which presents eco-innovation as any innovation that translates into an important step towards sustainable development, reducing the negative impact of production, strengthening nature’s resilience to human pressure, or using natural resources more efficiently and responsibly (Durante et al., 2019; European Commission, 2020). Similar to innovation eco-innovation can be viewed as multi and transdisciplinary (Bossle et al., 2016).

### Determinants of the implementation of the innovation strategy

**Fig. 1:**
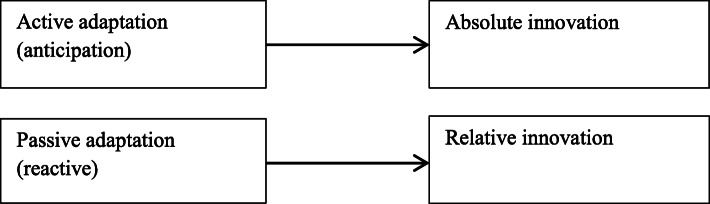
Relation between adaptation and innovation

Starting from the proposed division into continuous and jumping innovations, it is worth considering the determinants that stimulate and generate the implementation by enterprises of these types of innovations. Elements or determinants supporting and strengthening the innovation strategy are primarily the aforementioned adaptability of the company. Adaptive ability, i.e., the ability to adapt to changing conditions, is a key factor in undertaking innovative activities. The adaptive capacity of an enterprise depends on managers, their skills, professionalism, competence, openness, and financial resources that can be allocated to the R&D sphere and the purchase of new, expensive technologies. In research on the adaptability of energy companies, Borowski (2016, 2019) presents the adaptation matrix and divides adaptation into active (anticipative, prior, anterior) and passive (reactive) adaptation. If a company is able to apply anticipative/anterior adaptation, it will also have the ability to create and implement absolute innovations and will be able to introduce services, products, or technologies much earlier than competing companies. If a company has passive/reactive adaptability, it is a company that has the potential to implement imitative/relative innovations. The relationship between adaptation and innovation is presented in Fig. 1.

The ability to predict and anticipate market events gives the company the opportunity to create, initiate products, services, or processes that will be completely new on a global scale. On the other hand, the ability to adapt to existing or emerging solutions will allow the enterprise to implement innovations on a regional, local, or only scale of the enterprise.

### The role and significance of innovation and eco-innovation

The role and significance of innovation and eco-innovation for the development of enterprises results from their direct or indirect impact on the company’s competitiveness and its chances of gaining a significant position on the domestic or global market (Shankar & Narang, 2020; Zakrzewska-Bielawska, 2012) and innovation depend on the genre (type) of industries. Some branches can be called as creative industries, e.g., HighTech or biodegradable materials (Żelaziński et al., 2019; Zuhdi, 2015). Innovation may also increase the level of employment in the enterprise and affect efficiency (Okumu et al., 2019). The importance of innovation can be represented as follows:

- Innovations allow the introduction of products and technologies not yet available on the market, so they guarantee the company’s profitability
- Innovations facilitate the creation of a product, technological, or market niche for a company
- Leadership position even in a narrow area ensures competitive income
- Innovation creates a strong bargaining position in negotiations with customers
- The company’s prestige in the regional and national environment can be built on high innovation (Sosnowska & Łobejko, 2006)
- The basic principle of eco-innovation is to reduce the negative impact on the environment
- Eco-innovation is a widely accepted advantageous strategy when applied in a systematic, conscious, and advised way (Bossle et al., 2016)

### Research methods

Research on innovation in enterprises and research on new innovative solutions related to the use of bamboo in various industries have been carried out as quantitative and qualitative research. Therefore, the study was conducted on the basis of mixed-methods analysis. It is assumed that the use of a combination of quantitative and qualitative methods provides the possibility of greater flexibility in undertaking research, generating better-supported arguments based on research data and greater importance for a wider range of stakeholders. Using a mixed-methods approach (MMA), it is expected that the combination of quantitative and qualitative methods will eliminate the errors of individual methods and complement the results obtained at individual stages of the research process (Bazeley, 2015; Onwuegbuzie & Collins, 2007). The research methods used in the completed research are quantitative and qualitative methods (mixed-methods). Quantitative methods are presented by chi-squared test and V-Cramer contingency coefficient, while qualitative methods are presented by desk-research and primary research.

## Results

The results of the conducted research on the implementation of innovation in enterprises can be divided into two groups: results based on statistical methods and results based on literature review and surveys. The results show the role of employee groups in the creation of innovations inter alia; it was indicated that depending on the size of the company, different groups of employees are responsible for the process of initiating and creating innovation. The results show also that bamboo is a material with huge potential that affects the innovation of the company. The use of bamboo gives companies great development opportunities. More detailed and specific results are presented below.

### Quantitative research on business innovation

The research carried out by Baumann and Kritikos (2016) and Luengo-Valderrey and Moso-Díez (2019) showed the relationship between R&D and the size of the enterprise and results related to innovation as well as knowledge workers (Darroch & McNaughton, 2002) and their influence on company innovation. However, these own researches carried out and described here by the author complement the ideas initiated by mentioned researchers and explore the relationship between the size of the enterprise and the involvement of individual employee groups (knowledge group) in the innovation process. In quantitative research theoretical foundations are first identified. The theoretical foundations are built on the basis of a literature review and its critical analysis to determine the conceptual framework of the study by defining the concepts precisely. The quantitative approach in management sciences is based on statistics. Quantitative research was intended to show whether there is a relationship between the employee groups responsible for implementing innovation and the size of the company. Quantitative research carried out on a group of 560 enterprises^1^ (including 303 micro-enterprises, 62 mini, 85 medium, and 110 large) showed that the management is responsible for innovation (directors, managers), employees of a dedicated R&D team, and a group of other employees involved in innovation.

The average number of employees involved in innovation activities varies depending on the size of the company. In micro-enterprises conducting an innovative activity, it is on average 1 employee, in small 2, medium 6, and 17 large employees. This shows the obvious advantage of larger companies that can involve more human resources in innovation activities. In micro-firms, this is difficult because employees need to focus on ongoing activities. The number of responses on individual groups constituting sources of innovation is presented in Table 2.

**Table 2:** Individual employee groups that are a source of innovation

	CEO	R+D department	Others workers	Total
Micro company	215	33	67	315
Mini company	50	5	16	71
Medium company	60	21	31	112
Large company	90	39	43	72
Total	415	98	157	670

Source: own elaboration

Depending on the size of the company, it can be seen that the sources of innovation, involvement, and responsibility of individual employee groups in the innovation process are assessed by respondents at various levels. That is why it is important to ask whether there is a relationship between the size of the company and the involvement of these employee groups.

The chi-squared test was conducted to find the answer to this question. In chi-squared test, the null hypothesis H0 has been formulated: there is no relationship between the variables: the size of the enterprise and the groups responsible for implementing innovations. If the empirical chi-square is larger than the theoretical chi-square, the H_0_ hypothesis will be rejected and the H_1_ hypothesis that there is a relationship between these variables will be true (Tables 3 and 4).

$$ {x}_{emp}^2=\sum \limits_{i=1}^k\sum \limits_{j=1}^s\frac{{\left({n}_{ij}-{n}_{ij}^{theor}\right)}^2}{n_{ij}^{theor}} $$

**Table 3:** $$ {n}_{ij}^{theor} $$

	CEO	R+D department	Other workers
**Micro company**	${n}_{ij}^{theor}=\frac{315\ast 415}{670}=\mathrm{195,11}$	${n}_{ij}^{theor}=\frac{315\ast 98}{670}=46,07$	${n}_{ij}^{theor}=\frac{315\ast 157}{670}=73,82$
**Mini company**	${n}_{ij}^{theor}=\frac{71\ast 415}{670}=43,98$	${n}_{ij}^{theor}=\frac{71\ast 98}{670}=10,39$	${n}_{ij}^{theor}=\frac{71\ast 157}{670}=16,64$
**Medium company**	${n}_{ij}^{theor}=\frac{112\ast 415}{670}=69,37$	${n}_{ij}^{theor}=\frac{112\ast 98}{670}=16,38$	${n}_{ij}^{theor}=\frac{112\ast 157}{670}=26,24$
**Large company**	${n}_{ij}^{theor}=\frac{172\ast 415}{670}=\mathrm{106,54}$	${n}_{ij}^{theor}\frac{172\ast 98}{670}=25,16$	${n}_{ij}^{theor}=\frac{172\ast 157}{670}=40,30$

Source: own elaboration

**Table 4:** Chi-squared empirical ${\chi}_{emp}^2$

	CEO	R+D department	Other workers
**Micro company**	${x}_{emp}^2=\frac{{\left(215-\mathrm{195,11}\right)}^2}{\mathrm{195,11}}=2,03$	${x}_{emp}^2=\frac{{\left(33-46,07\right)}^2}{46,07}=3,71$	${x}_{emp}^2=\frac{{\left(67-73,82\right)}^2}{73,82}=0,63$
**Mini company**	${x}_{emp}^2=\frac{{\left(51-43,98\right)}^2}{43,98}=1,12$	${x}_{emp}^2=\frac{{\left(4-10,39\right)}^2}{10,39}=3,93$	${x}_{emp}^2=\frac{{\left(16-16,64\right)}^2}{16,64}=0,03$
**Medium company**	${x}_{emp}^2=\frac{{\left(60-69,37\right)}^2}{69,37}=1,27$	${x}_{emp}^2=\frac{{\left(21-16,38\right)}^2}{16,38}=1,30$	${x}_{emp}^2=\frac{{\left(31-26,24\right)}^2}{26,24}=0,86$
**Large company**	${x}_{emp}^2=\frac{{\left(89-\mathrm{106,54}\right)}^2}{\mathrm{106,54}}=2,89$	${x}_{emp}^2=\frac{{\left(40-25,16\right)}^2}{25,16}=8,75$	${x}_{emp}^2=\frac{{\left(43-40,30\right)}^2}{40,30}=0,18$
	∑=7,31	∑=17,69	∑=1,7
	${\boldsymbol{\chi}}_{\boldsymbol{emp}}^{\mathbf{2}}$**=26,70**		

Source: own elaboration

After calculating the empirical value ${\chi}_{emp}^2$, it must be compared with the theoretical value ${\chi}_{theor}^2$ in the table. To do this, first determine the number of degrees of freedom and then read the tabular value assuming the confidence level alpha level of significance *α* = 0.05. In our case, degrees of freedom df=(*c*-1)*(*r*-*1*)=(3-1)*(4-1)=6 where *c* – column and *r* – row (Table 5).

**Table 5:** The value of chi square theoretical ${\chi}_{theor}^2$

Alpha level of significance									
**df**	0.20	0.10	0.05	0.025	0.02	0.01	0.005	0.002	0.001
**1**	1.642	2.706	3.841	5.024	5.412	6.635	7.879	9.550	10.828
**2**	3.219	4.605	5.991	7.378	7.824	9.210	10.597	12.429	13.816
**3**	4.642	6.251	7.815	9.348	9.837	11.345	12.838	14.796	16.266
**4**	5.989	7.779	9.488	11.143	11.668	13.277	14.860	16.924	18.467
**5**	7.289	9.236	11.070	12.833	13.388	15.086	16.750	18.907	20.515
**6**	8.558	10.645	**12.592**	14.449	15.033	16.812	18.548	20.791	22.458
**7**	9.803	12.017	14.067	16.013	16.622	18.475	20.278	22.601	24.322

*χ*^emp^=26,70

*χ*^theoret^=12,592

$$ {\chi}_{emp}>{\chi}_{theoret} $$

So null hypothesis H0 was rejected. There is an association between variables. The results of the chi-squared test have shown that there is a relationship between the size of the enterprise and the groups involved and responsible for implementing the innovation. After conducting the chi-squared test, which pointed to the existence of relationships, it is worth asking the question about the strength of these relationships. The answer for the strength of dependence can be obtained by calculating the V-Cramer contingency coefficient.

$$ V=\sqrt{\frac{X^2}{n\left(m-1\right)}\kern0.5em }=\sqrt{\frac{26,{70}^2}{560\left(3-1\right)}}=0,7978 $$

The coefficient was 0.7978 which means that there is a strong relationship between the variables. Depending on the size of the company, different groups are responsible for the process of initiating and creating innovation. In most of the surveyed enterprises, creating innovation rests on the shoulders of the management board and top management, but in large enterprises and corporations as well as in medium-sized companies, creative employees from outside the R&D team also have a significant impact on initiating innovation. Comparing large and medium-sized enterprises with micro and mini enterprises, it can be seen that R&D teams in the latter play a much smaller role than in large and medium-sized companies, which probably results from the fact that maintaining a R&D team requires large financial outlays.

### Qualitative research on the use of bamboo by enterprises from various industries

In order to learn the directions of the innovation strategy of enterprises, research questions were formulated and empirical research (observations and deep interviews) was carried out, which allowed verification of research questions and make a generalization. Qualitative research is characterized by a method, in which a priori image of reality is not assumed, but research questions are formulated and then generalizations are formulated on the basis of empirical research. Qualitative research is used to generalize the understanding of occurring phenomena. Qualitative research is not presented in numbers (in quantities) but concerns the characteristics of the phenomena studied. They focus on identifying facts, and most often answer questions why, what, how much, how strongly, how often, and in what part.

Then, qualitative research was to show the motives for implementing innovation on the use of bamboo on an industrial scale. Research on the use of bamboo in various branches of the economy has shown wide possibilities of using bamboo resulting from its advantages. Bamboo due to its physicochemical properties can be used in the construction, textile, food, and energy industries.

Primary research on issues of innovative bamboo applications was carried out as the interviews with the heads of companies using bamboo as their basic material. Interviews took place in January–February 2020. Interviews were conducted by an Internet survey according to the script, and the questions posted in the questionnaire are presented below:

1. In which country does your company/business exist?
2. How old is your company?
3. How many workers do you have in your company?
4. Who is responsible for innovation in your company?
5. How long do you use bamboo as a product material in your company?
6. Why did you start to use bamboo?
7. Is it a profitable activity to use bamboo, to work with bamboo?
8. Is bamboo your basic material/basic activity?
9. Do you have other activities? Which one?
10. Are there any competitors around your business?
11. How can you describe you’re your experience with bamboo?
12. On which market do you exist? Local, national (domestic), international?
13. Do you invest in Research and Development (R&D) on the bamboo subject?
14. How many percentages of your assets do you invest in R+D concerning bamboo?
15. Did you start to use bamboo from the beginning of your business or you added bamboo gradually, step by step?
16. Did you follow the customers’ needs, or you anticipated in their needs/expectations?

The results of qualitative research conducted on a group of companies from various sectors of the economy (energy, water equipment, automotive, and textile) have been analyzed by the author. The answers provided allowed for drawing general conclusions regarding innovation based on the use of bamboo. The responses collected from the companies showed that bamboo is a material with huge potential that affects the innovation of the company. The use of bamboo gives companies great development opportunities.

### Innovation in the energy sector

Research results published in 2019 regarding enterprises from the energy market showed that companies should develop the R&D sphere, adapt to the requirements of the environment, and implement innovations (Borowski, 2019). The conclusions and recommendations made in the mentioned study showed the need to continue research towards innovative and eco-innovative solutions. Enterprises should also pay attention to the impact on the environment and look for innovative solutions that reduce the negative impact, which can be described as eco-efficiency. Eco-efficiency is a “win–win” business strategy, which helps companies save money and reduce their negative environmental impact. Eco-efficiency and production costs are therefore important for the management of the enterprise and obtained energy efficiency and unit consumption indicators can be used to define environmental standards. Eco-efficiency is closely related to efficiency consisting in achieving high environmental results in reducing the consumption of natural resources, reducing the emission of substances polluting the environment, and reducing the mass of generated waste (Prasad et al., 2004). Eco-efficiency is often pursued through approaches and “tools” such as cleaner production, environmental management systems, life cycle assessment (LCA), and environmental design. These tools help companies identify opportunities to improve resource efficiency and reduce environmental impact. Due to the increasing consumption of materials, social pressure on environmental issues, stricter regulations, and a higher level of competition, companies will have to take environmental considerations into account in their strategic planning and gradually switch to a circular economy. The circular economy (CE) will significantly contribute to achieving climate neutrality in the future (Maranesi & De Giovanni, 2020) and will contribute to attain higher performance on business activities. Eco-efficiency means moreover increasing process efficiencies and reducing environmental impact, for example by reducing the material intensity of goods and services, by reducing the energy intensity of goods and services, by reducing toxic emissions, by maximizing the use of renewable resources, and enhancing the material recyclability. CE represents an enormous opportunity to integrate sustainability into the enterprises’ vision and reconcile economic, environmental, and social goals. CE as a significant and important issue was considered in recent years by many researchers. Elia (2017) indicated that CE can be a kind of path to increase the sustainability of the economic system. An important role will play reuse, repair, and recycle and become crucial activities in many sectors, whereas Korhonen (2018) noticed that CE attracts both the business community and the community of decision-makers to work for sustainable development. Roos Lindgreen, Salomone, and Reyes (2020) mentioned that enterprises have implemented CE strategies to reduce their resource use and its associated impacts in order to boost economic competitiveness and generate positive social impact.

Enterprises operate in changing conditions and various elements influence the undertaking of innovative activities to varying degrees. For energy companies, one of the most important elements of the macro environment is the ecological environment, which brings with it many tight requirements for the broadly understood environment. Enterprises very often are looking for eco-friendly, proactive environmental strategies, which bring the eco-efficiency (Magarey et al., 2019; Seroka-Stolka & Fijorek, 2020). Due to the requirements of the ecological environment, energy companies should move towards reducing pollution, lowering emissions, and reducing negative impacts on the environment. The problems of climate change, in addition to constantly rising energy costs and depleting fossil fuel resources, make it necessary to look for new, ecological, widely available and efficient methods of energy production. Renewable energy using wind, the sun, water and biomass resources is leading in this respect. Therefore, companies should look for alternative raw materials and energy materials for coal. One of such solutions may be the use of renewable biomass from bamboo instead of wood biomass used so far. Bamboo biomass is characterized by rapid growth and increase of wood mass as well as slight interference in the environment.

Bamboo is a sustainable and environmentally superior material (Ogawa et al., 2016). Bamboo can be potential as bioenergetic material. Bamboo has good fiber quality and has many important fuel properties, including bioenergy substances such as low ash content and alkaline index. The main components of ash arising from the combustion of bamboo are potassium dioxide (K_2_O) and silica (SiO_2_). Ashes also contain chlorine (Cl), calcium (CaO), and magnesium (MgO) (Kumar & Chandrashekar, 2014). To assess bamboo quality as a biofuel, knowledge of chlorine (Cl) and sulfur (S) content is also needed. A large amount of these elements causes corrosion and contamination of boilers, pipes, supply lines, and an increase in SOx, Cl_2_, and HCl emissions. The ash content is from 1.7 to 5% which is a good result compared to other biomaterials which is rice husk, which after burning contains 17% ash.

As bamboo quality and growth improves through systematic shearing, bamboo can be harvested every three years without damaging the environment. For example, if we compare the average life expectancy of a redwood, which is about 500 years, with a bamboo plant, we can see that bamboo can be harvested and reborn over 150 times at the same time. The energy produced from bamboo biomass has great potential and can become an alternative to traditional fossil fuels. Bamboo biomass comes from stems, branches, and leaves. Bamboo biomass can be transformed in many different ways (biochemical or thermal conversion) to produce different energy products (synthesis gas and biofuels or charcoal) that can become a substitute for existing fossil fuels. It should be noted, however, that the use of bamboo biomass alone is not able to meet global energy demand. Therefore, it must combine with other sources to make the best use of their potential and ensure sustainable energy supplies (Le & Truong, 2014). Bamboo biomass is characterized by a relatively higher calorific value than other biomass, which means that it is a good material for direct combustion (e.g., co-combustion in a thermal power plant). Many different projects on the use of energy from bamboo operate, operate and are implemented worldwide. In African countries, bamboo biomass is very popular and most often used to replace firewood or the production of charcoal for home use. An innovative solution in the energy sector is a power plant entirely fired with bamboo mass. In Japan, a large-scale biomass power plant construction project using bamboo alone is underway. This type of power plant is the first in the world. A comprehensive power plant system is being developed, ranging from cutting, crushing, and burning bamboo to generating electricity. The power plant overcame problems caused by minerals contained in bamboo during combustion, including reduced energy production efficiency and boiler problems. Energy market companies adapt to market requirements and environmental requirements by implement innovative solutions. Companies that have partially or fully switched to energy production from bamboo have become environmentally friendly, and they have adopted a strategy of eco-technological innovation.

### Innovations in the water equipment industry

The innovative company that introduced product innovations regarding bamboo structures in surfboards is a well-known company of Gary Young. The company was looking for new solutions that would be more environmentally friendly. For a surfboard company to become eco-innovative and move into a circular economy, it should stop using raw materials based on nasty chemicals, fumes, waste, non-recyclable, and insufficiently durable. The company should use materials that are durable, ecological, and reusable. The company began looking for natural materials that can replace the artificial fibers used so far. Gary Young’s company was looking for solutions to limit the harmful effects on the surrounding environment. Bamboo turned out to be such a material. Thanks to its mechanical properties, bamboo can replace glass fibers, which are relatively expensive. Bamboo has the highest strength-to-weight ratio of any natural fiber. Due to those properties such as durability and repairability of bamboo surfboard, we can conclude that those factors are important aspects of a green board. The longer a board can be surfed and repaired, without losing performance, the more eco-friendly it is. According to Gary Young, bamboo can be a cheap and ecological replacement for carbon fiber. Bamboo absorbs very well the strength of strong impacts. The production process is cheap and environmentally friendly. Therefore, the eco surfboard technology, based on bamboo construction, became an innovative solution.

### Innovations in the automotive industry

Innovation in the automotive industry signifies various products and process development, developed new perspectives, and useful concepts (Holtskog, 2017). In recent years, innovations related to zero-emission cars, i.e., electric cars (Kupczyk et al., 2020; Saritas et al., 2019) are popular, but the reduction of harmful effects on the environment can also be implemented by including the production of bamboo elements. Automotive companies increasingly use bamboo as part of the car interior or as parts of usable parts, e.g., Lexus CT uses bamboo in installed speakers to increase and enlargement the quality and timbre of the sound. In addition, Lexus is moving towards new technologies based on renewable sources to replace, where possible, plastic and fiberglass parts and components with bamboo elements, e.g., steering wheel, dashboard. Lexus’ innovation strategy can be classified as technological innovation as well as eco-innovation. This is due to the fact that new technical solutions have been implemented to improve the comfort of using the car with the use of environmentally friendly materials.

### Innovations in the textile industry

Hazardous substances from textile production affect direct the health of both textile workers and wearers of clothes, and they escape into the environment. Some clothes, during the washing process, release plastic microfibers, of which around half a million tons every year contribute to ocean pollution, and then, living organisms are contaminated (Borowski, 2017; Ellen MacArthur Foundation, 2017). In a new textile economy, in which replacement of petrochemical-based synthetic fibers was realized, to organic plants (bamboo), an eco-innovative solution took place. Based on the principles of a circular economy (CE), clothes, textiles, and fibers produced from bamboo are kept at their highest value during use and re-enter the economy afterwards, never-ending up as waste. Products made from bamboo are often labeled as “eco-friendly”, “biodegradable”, and “antimicrobial” irrespective of their method of manufacturing, so they are suitable materials to work under the CE regime (Nayak & Mishra, 2016). With a specific emphasis on innovation towards a technological and product innovation, a new textile economy presents an opportunity to deliver substantially better economic, societal, and environmental outcomes (Ellen MacArthur Foundation, 2017).

#### A company that produce socks

A textile company that produces bamboo fiber socks. Bamboo and cotton are very good materials. They are breathable, allow the skin to breathe, but bamboo is superior to cotton in that much less chemicals are used when growing bamboo because bamboo is more resistant to pests and parasites, has antibacterial properties, so its use is more beneficial for the environment. In addition, bamboo socks do not compress the ankles, perfectly keep heat, and are very durable. Moreover, bamboo socks offer moisture-wicking properties and superior comfort. In the production of socks, their impact on thromboembolism disease is important. Many socks can have negative effects on blood circulation (Zadow et al., 2018).

#### A company that produces towels

In 2009, the company launched an innovative fabric production, with 100% bamboo yarn. The company has achieved a higher level of quality through the use of bamboo fibers. The company began to create luxury products that met the expectations of the most demanding customers. The bamboo yarn has natural antibacterial properties, thanks to which bamboo towels are perfect for contact with sensitive skin and in an environment exposed to the formation of bacteria. Very good hygroscopic properties of the raw material ensure excellent absorbency and short drying time of the products. Thanks to the unique technology products have the highest absorbency and softness. Raw materials developed in laboratories, appropriate air humidity, perfectly selected thermal treatments and an extended process of finishing the product provide towels production with excellent performance parameters. The company also has created a bamboo-line collection, which includes fabrics, towels, bedding, shirts, and even nappies for children and babies.

Both companies mentioned above have applied product innovations and technological innovations. Moreover, thanks to the application of environment-friendly solutions, these innovations can be classified as eco-innovations, which can also create an element of the circular economy.

## Conclusion

The results of the conducted research can be shared into two main groups, which interpenetrate and complement each other. The first group of the results shows that the innovation strategy is a drive for the creative functioning of enterprises. In most enterprises, the initiator of innovation is the management, while R&D teams are an important source of innovation primarily in large and medium-sized companies.

The second group of results shows the implementation of innovation strategies, mainly technological and product-related, in enterprises belonging to various sectors of the economy. Thanks to the use of bamboo as an ecological material; it is possible to implement eco-innovations and join the closed-loop economy. Innovations allow enterprises to maintain a proper competitive position on the market and in many sectors of the economy the innovative use of bamboo gives a competitive advantage and also positively affects the environment. The use of bamboo is a significant and important fact in the current climate change situation. The wide use of bamboo will allow companies to develop further with a positive impact on the surrounding environment and switch on from unsustainable development to one of sustainable development by using eco-efficiency.

The limitations of the research refer to the conducted surveys, which in the future would be worth carrying out on a larger research sample. The group of companies participating in future research should represent most sectors of the economy. It would also be worth analyzing how the COVID-19 pandemic influenced the development of innovation, which sectors developed faster and which developed slower.

## Data Availability

Not applicable.

## Footnotes

1 Report from the research “Monitoring innowacyjności polskich przedsiębiorstw” carried out on behalf of the Polish Agency for Enterprise Development, Warsaw 2019. http://www.parp.gov.pl
